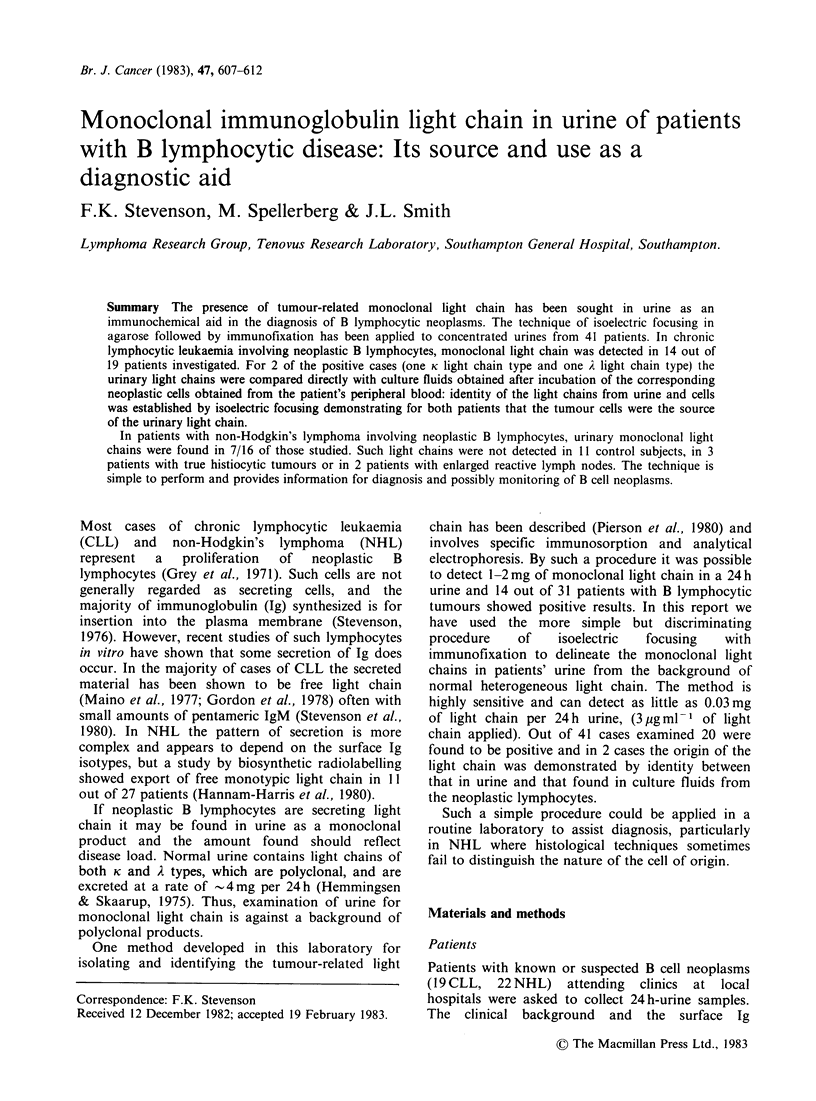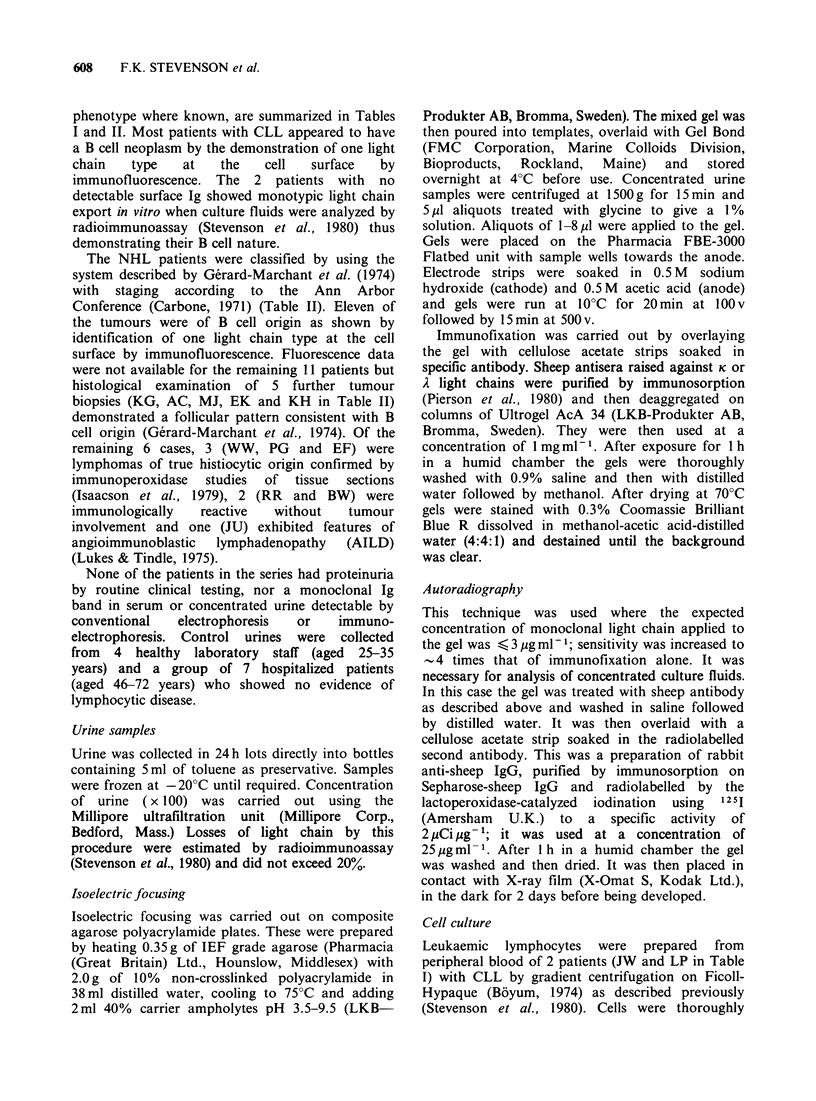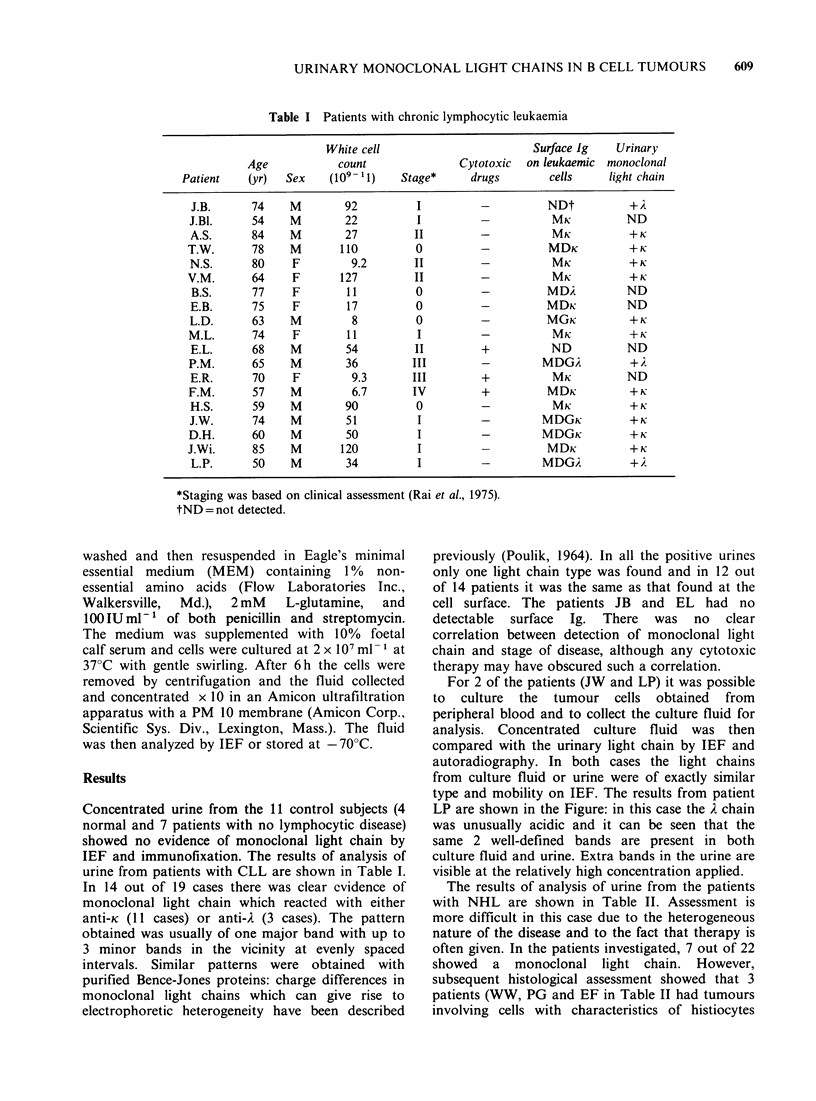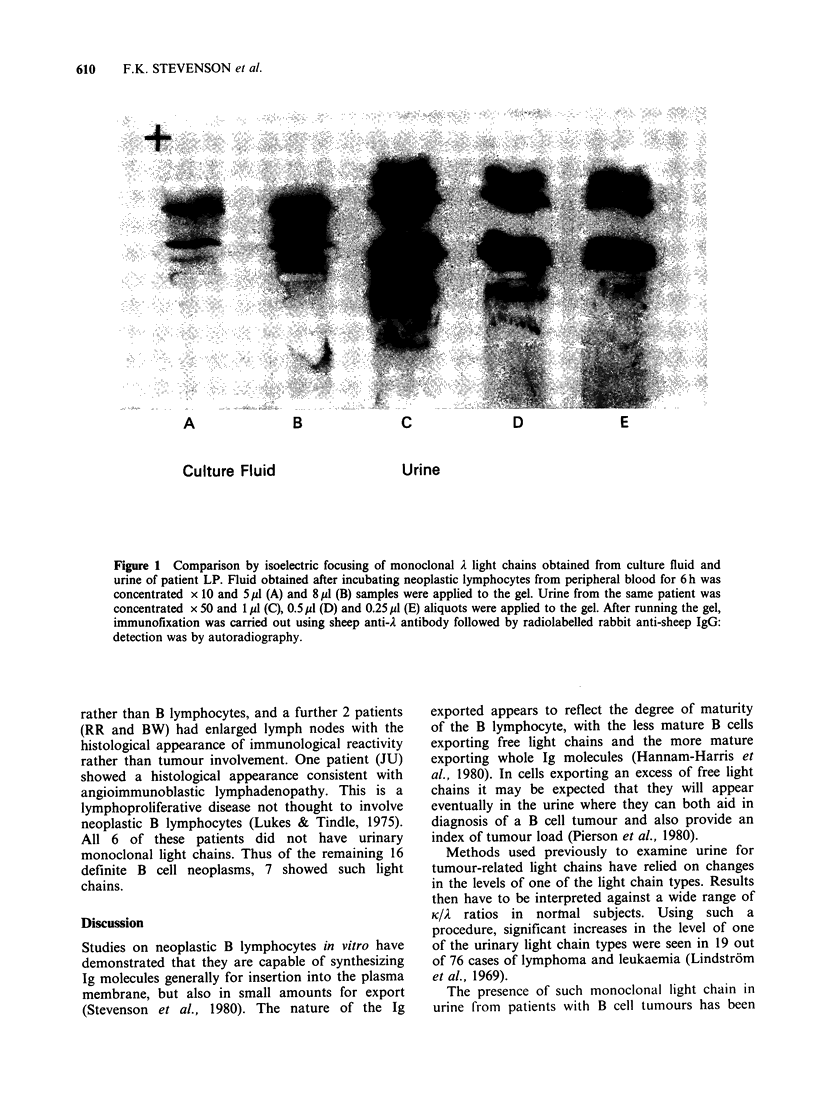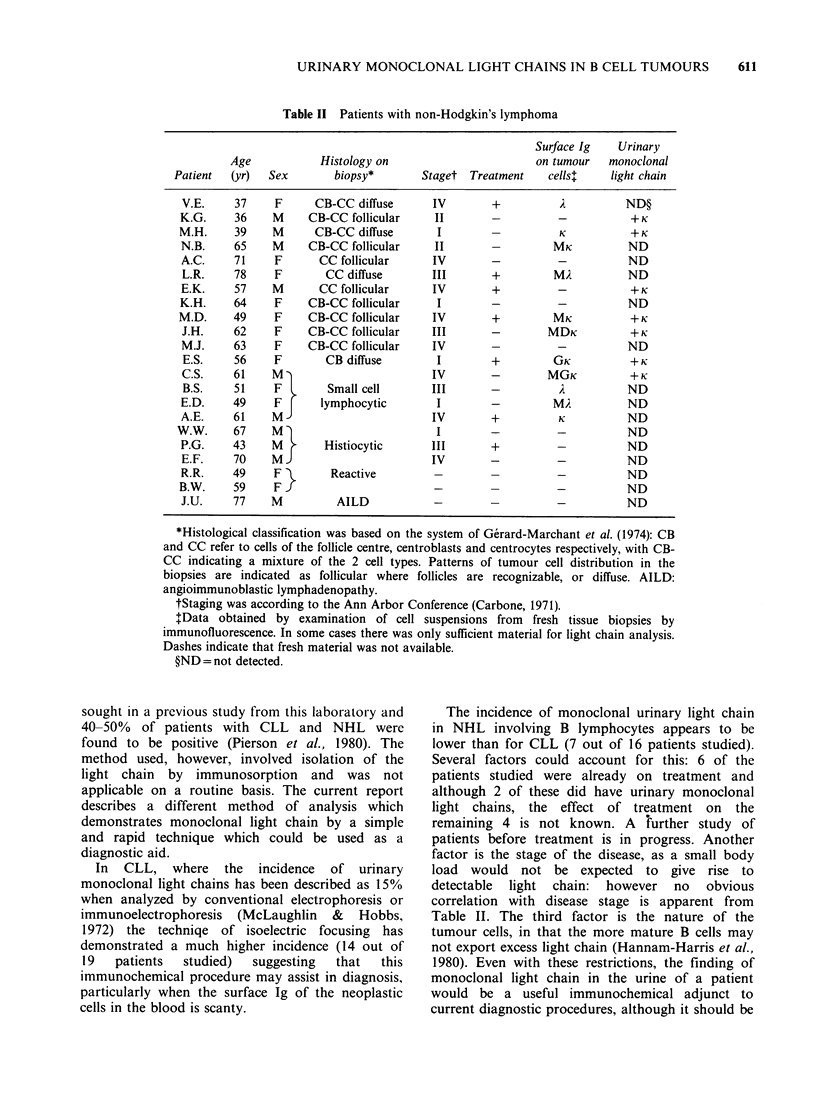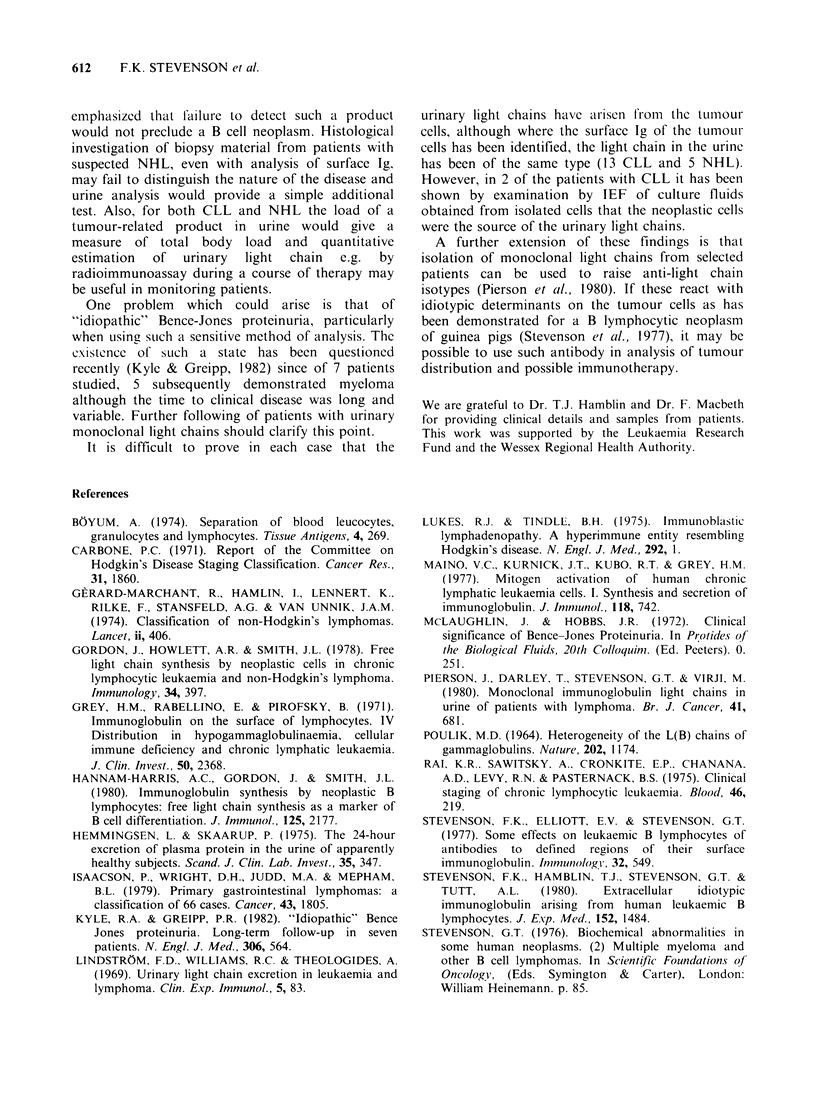# Monoclonal immunoglobulin light chain in urine of patients with B lymphocytic disease: its source and use as a diagnostic aid.

**DOI:** 10.1038/bjc.1983.98

**Published:** 1983-05

**Authors:** F. K. Stevenson, M. Spellerberg, J. L. Smith

## Abstract

**Images:**


Br. J. Cancer (1983), 47, 607–612

### Monoclonal immunoglobulin light chain in urine of patients with B lymphocytic disease: Its source and use as a diagnostic aid

F.K. Stevenson, M. Spellerberg & J.L. Smith

*Lymphoma Research Group, Tenovus Research Laboratory, Southampton General Hospital, Southampton.*

**Summary** The presence of tumour-related monoclonal light chain has been sought in urine as an immunochemical aid in the diagnosis of B lymphocytic neoplasms. The technique of isoelectric focusing in agarose followed by immunofixation has been applied to concentrated urines from 41 patients. In chronic lymphocytic leukaemia involving neoplastic B lymphocytes, monoclonal light chain was detected in 14 out of 19 patients investigated. For 2 of the positive cases (one $\kappa$ light chain type and one $\lambda$ light chain type) the urinary light chains were compared directly with culture fluids obtained after incubation of the corresponding neoplastic cells obtained from the patient's peripheral blood: identity of the light chains from urine and cells was established by isoelectric focusing demonstrating for both patients that the tumour cells were the source of the urinary light chain.

In patients with non-Hodgkin's lymphoma involving neoplastic B lymphocytes, urinary monoclonal light chains were found in 7/16 of those studied. Such light chains were not detected in 11 control subjects, in 3 patients with true histiocytic tumours or in 2 patients with enlarged reactive lymph nodes. The technique is simple to perform and provides information for diagnosis and possibly monitoring of B cell neoplasms.

Most cases of chronic lymphocytic leukaemia (CLL) and non-Hodgkin's lymphoma (NHL) represent a proliferation of neoplastic B lymphocytes (Grey *et al.*, 1971). Such cells are not generally regarded as secreting cells, and the majority of immunoglobulin (Ig) synthesized is for insertion into the plasma membrane (Stevenson, 1976). However, recent studies of such lymphocytes *in vitro* have shown that some secretion of Ig does occur. In the majority of cases of CLL the secreted material has been shown to be free light chain (Maino *et al.*, 1977; Gordon *et al.*, 1978) often with small amounts of pentameric IgM (Stevenson *et al.*, 1980). In NHL the pattern of secretion is more complex and appears to depend on the surface Ig isotypes, but a study by biosynthetic radiolabelling showed export of free monotypic light chain in 11 out of 27 patients (Hannam-Harris *et al.*, 1980).

If neoplastic B lymphocytes are secreting light chain it may be found in urine as a monoclonal product and the amount found should reflect disease load. Normal urine contains light chains of both $\kappa$ and $\lambda$ types, which are polyclonal, and are excreted at a rate of ~4 mg per 24 h (Hemmingsen & Skaarup, 1975). Thus, examination of urine for monoclonal light chain is against a background of polyclonal products.

One method developed in this laboratory for isolating and identifying the tumour-related light chain has been described (Pierson *et al.*, 1980) and involves specific immunosorption and analytical electrophoresis. By such a procedure it was possible to detect 1–2 mg of monoclonal light chain in a 24 h urine and 14 out of 31 patients with B lymphocytic tumours showed positive results. In this report we have used the more simple but discriminating procedure of isoelectric focusing with immunofixation to delineate the monoclonal light chains in patients' urine from the background of normal heterogeneous light chain. The method is highly sensitive and can detect as little as 0.03 mg of light chain per 24 h urine, ($3\,\mu g\,ml^{-1}$ of light chain applied). Out of 41 cases examined 20 were found to be positive and in 2 cases the origin of the light chain was demonstrated by identity between that in urine and that found in culture fluids from the neoplastic lymphocytes.

Such a simple procedure could be applied in a routine laboratory to assist diagnosis, particularly in NHL where histological techniques sometimes fail to distinguish the nature of the cell of origin.

### Materials and methods

### *Patients*

Patients with known or suspected B cell neoplasms (19 CLL, 22 NHL) attending clinics at local hospitals were asked to collect 24 h-urine samples. The clinical background and the surface Ig

Correspondence: F.K. Stevenson
Received 12 December 1982; accepted 19 February 1983.

© The Macmillan Press Ltd., 1983

phenotype where known, are summarized in Tables I and II. Most patients with CLL appeared to have a B cell neoplasm by the demonstration of one light chain type at the cell surface by immunofluorescence. The 2 patients with no detectable surface Ig showed monotypic light chain export *in vitro* when culture fluids were analyzed by radioimmunoassay (Stevenson *et al.*, 1980) thus demonstrating their B cell nature.

The NHL patients were classified by using the system described by Gérard-Marchant *et al.* (1974) with staging according to the Ann Arbor Conference (Carbone, 1971) (Table II). Eleven of the tumours were of B cell origin as shown by identification of one light chain type at the cell surface by immunofluorescence. Fluorescence data were not available for the remaining 11 patients but histological examination of 5 further tumour biopsies (KG, AC, MJ, EK and KH in Table II) demonstrated a follicular pattern consistent with B cell origin (Gérard-Marchant *et al.*, 1974). Of the remaining 6 cases, 3 (WW, PG and EF) were lymphomas of true histiocytic origin confirmed by immunoperoxidase studies of tissue sections (Isaacson *et al.*, 1979), 2 (RR and BW) were immunologically reactive without tumour involvement and one (JU) exhibited features of angioimmunoblastic lymphadenopathy (AILD) (Lukes & Tindle, 1975).

None of the patients in the series had proteinuria by routine clinical testing, nor a monoclonal Ig band in serum or concentrated urine detectable by conventional electrophoresis or immuno-electrophoresis. Control urines were collected from 4 healthy laboratory staff (aged 25–35 years) and a group of 7 hospitalized patients (aged 46–72 years) who showed no evidence of lymphocytic disease.

*Urine samples*

Urine was collected in 24 h lots directly into bottles containing 5 ml of toluene as preservative. Samples were frozen at $-20$°C until required. Concentration of urine ($\times 100$) was carried out using the Millipore ultrafiltration unit (Millipore Corp., Bedford, Mass.) Losses of light chain by this procedure were estimated by radioimmunoassay (Stevenson *et al.*, 1980) and did not exceed 20%.

*Isoelectric focusing*

Isoelectric focusing was carried out on composite agarose polyacrylamide plates. These were prepared by heating 0.35 g of IEF grade agarose (Pharmacia (Great Britain) Ltd., Hounslow, Middlesex) with 2.0 g of 10% non-crosslinked polyacrylamide in 38 ml distilled water, cooling to 75°C and adding 2 ml 40% carrier ampholytes pH 3.5–9.5 (LKB—Produkter AB, Bromma, Sweden). The mixed gel was then poured into templates, overlaid with Gel Bond (FMC Corporation, Marine Colloids Division, Bioproducts, Rockland, Maine) and stored overnight at 4°C before use. Concentrated urine samples were centrifuged at 1500 g for 15 min and 5 $\mu$l aliquots treated with glycine to give a 1% solution. Aliquots of 1–8 $\mu$l were applied to the gel. Gels were placed on the Pharmacia FBE-3000 Flatbed unit with sample wells towards the anode. Electrode strips were soaked in 0.5 M sodium hydroxide (cathode) and 0.5 M acetic acid (anode) and gels were run at 10°C for 20 min at 100 v followed by 15 min at 500 v.

Immunofixation was carried out by overlaying the gel with cellulose acetate strips soaked in specific antibody. Sheep antisera raised against $\kappa$ or $\lambda$ light chains were purified by immunosorption (Pierson *et al.*, 1980) and then deaggregated on columns of Ultrogel AcA 34 (LKB-Produkter AB, Bromma, Sweden). They were then used at a concentration of 1 mg ml$^{-1}$. After exposure for 1 h in a humid chamber the gels were thoroughly washed with 0.9% saline and then with distilled water followed by methanol. After drying at 70°C gels were stained with 0.3% Coomassie Brilliant Blue R dissolved in methanol-acetic acid-distilled water (4:4:1) and destained until the background was clear.

*Autoradiography*

This technique was used where the expected concentration of monoclonal light chain applied to the gel was $\leqslant 3\ \mu$g ml$^{-1}$; sensitivity was increased to $\sim$4 times that of immunofixation alone. It was necessary for analysis of concentrated culture fluids. In this case the gel was treated with sheep antibody as described above and washed in saline followed by distilled water. It was then overlaid with a cellulose acetate strip soaked in the radiolabelled second antibody. This was a preparation of rabbit anti-sheep IgG, purified by immunosorption on Sepharose-sheep IgG and radiolabelled by the lactoperoxidase-catalyzed iodination using $^{125}$I (Amersham U.K.) to a specific activity of 2 $\mu$Ci $\mu$g$^{-1}$; it was used at a concentration of 25 $\mu$g ml$^{-1}$. After 1 h in a humid chamber the gel was washed and then dried. It was then placed in contact with X-ray film (X-Omat S, Kodak Ltd.), in the dark for 2 days before being developed.

*Cell culture*

Leukaemic lymphocytes were prepared from peripheral blood of 2 patients (JW and LP in Table I) with CLL by gradient centrifugation on Ficoll-Hypaque (Böyum, 1974) as described previously (Stevenson *et al.*, 1980). Cells were thoroughly

**Table I** Patients with chronic lymphocytic leukaemia

| *Patient* | *Age (yr)* | *Sex* | *White cell count* ($10^{9-1}$l) | *Stage** | *Cytotoxic drugs* | *Surface Ig on leukaemic cells* | *Urinary monoclonal light chain* |
|---|---|---|---|---|---|---|---|
| J.B. | 74 | M | 92 | I | – | ND† | +λ |
| J.Bl. | 54 | M | 22 | I | – | Mκ | ND |
| A.S. | 84 | M | 27 | II | – | Mκ | +κ |
| T.W. | 78 | M | 110 | 0 | – | MDκ | +κ |
| N.S. | 80 | F | 9.2 | II | – | Mκ | +κ |
| V.M. | 64 | F | 127 | II | – | Mκ | +κ |
| B.S. | 77 | F | 11 | 0 | – | MDλ | ND |
| E.B. | 75 | F | 17 | 0 | – | MDκ | ND |
| L.D. | 63 | M | 8 | 0 | – | MGκ | +κ |
| M.L. | 74 | F | 11 | I | – | Mκ | +κ |
| E.L. | 68 | M | 54 | II | + | ND | ND |
| P.M. | 65 | M | 36 | III | – | MDGλ | +λ |
| E.R. | 70 | F | 9.3 | III | + | Mκ | ND |
| F.M. | 57 | M | 6.7 | IV | + | MDκ | +κ |
| H.S. | 59 | M | 90 | 0 | – | Mκ | +κ |
| J.W. | 74 | M | 51 | I | – | MDGκ | +κ |
| D.H. | 60 | M | 50 | I | – | MDGκ | +κ |
| J.Wi. | 85 | M | 120 | I | – | MDκ | +κ |
| L.P. | 50 | M | 34 | I | – | MDGλ | +λ |

*Staging was based on clinical assessment (Rai *et al.*, 1975).
†ND = not detected.

washed and then resuspended in Eagle's minimal essential medium (MEM) containing 1% non-essential amino acids (Flow Laboratories Inc., Walkersville, Md.), 2 mM L-glutamine, and 100 IU ml$^{-1}$ of both penicillin and streptomycin. The medium was supplemented with 10% foetal calf serum and cells were cultured at $2 \times 10^7$ ml$^{-1}$ at 37°C with gentle swirling. After 6 h the cells were removed by centrifugation and the fluid collected and concentrated ×10 in an Amicon ultrafiltration apparatus with a PM 10 membrane (Amicon Corp., Scientific Sys. Div., Lexington, Mass.). The fluid was then analyzed by IEF or stored at −70°C.

### Results

Concentrated urine from the 11 control subjects (4 normal and 7 patients with no lymphocytic disease) showed no evidence of monoclonal light chain by IEF and immunofixation. The results of analysis of urine from patients with CLL are shown in Table I. In 14 out of 19 cases there was clear evidence of monoclonal light chain which reacted with either anti-κ (11 cases) or anti-λ (3 cases). The pattern obtained was usually of one major band with up to 3 minor bands in the vicinity at evenly spaced intervals. Similar patterns were obtained with purified Bence-Jones proteins: charge differences in monoclonal light chains which can give rise to electrophoretic heterogeneity have been described previously (Poulik, 1964). In all the positive urines only one light chain type was found and in 12 out of 14 patients it was the same as that found at the cell surface. The patients JB and EL had no detectable surface Ig. There was no clear correlation between detection of monoclonal light chain and stage of disease, although any cytotoxic therapy may have obscured such a correlation.

For 2 of the patients (JW and LP) it was possible to culture the tumour cells obtained from peripheral blood and to collect the culture fluid for analysis. Concentrated culture fluid was then compared with the urinary light chain by IEF and autoradiography. In both cases the light chains from culture fluid or urine were of exactly similar type and mobility on IEF. The results from patient LP are shown in the Figure: in this case the λ chain was unusually acidic and it can be seen that the same 2 well-defined bands are present in both culture fluid and urine. Extra bands in the urine are visible at the relatively high concentration applied.

The results of analysis of urine from the patients with NHL are shown in Table II. Assessment is more difficult in this case due to the heterogeneous nature of the disease and to the fact that therapy is often given. In the patients investigated, 7 out of 22 showed a monoclonal light chain. However, subsequent histological assessment showed that 3 patients (WW, PG and EF in Table II had tumours involving cells with characteristics of histiocytes

**Figure 1** Comparison by isoelectric focusing of monoclonal λ light chains obtained from culture fluid and urine of patient LP. Fluid obtained after incubating neoplastic lymphocytes from peripheral blood for 6 h was concentrated ×10 and 5 μl (A) and 8 μl (B) samples were applied to the gel. Urine from the same patient was concentrated ×50 and 1 μl (C), 0.5 μl (D) and 0.25 μl (E) aliquots were applied to the gel. After running the gel, immunofixation was carried out using sheep anti-λ antibody followed by radiolabelled rabbit anti-sheep IgG: detection was by autoradiography.

rather than B lymphocytes, and a further 2 patients (RR and BW) had enlarged lymph nodes with the histological appearance of immunological reactivity rather than tumour involvement. One patient (JU) showed a histological appearance consistent with angioimmunoblastic lymphadenopathy. This is a lymphoproliferative disease not thought to involve neoplastic B lymphocytes (Lukes & Tindle, 1975). All 6 of these patients did not have urinary monoclonal light chains. Thus of the remaining 16 definite B cell neoplasms, 7 showed such light chains.

### Discussion

Studies on neoplastic B lymphocytes *in vitro* have demonstrated that they are capable of synthesizing Ig molecules generally for insertion into the plasma membrane, but also in small amounts for export (Stevenson *et al.*, 1980). The nature of the Ig exported appears to reflect the degree of maturity of the B lymphocyte, with the less mature B cells exporting free light chains and the more mature exporting whole Ig molecules (Hannam-Harris *et al.*, 1980). In cells exporting an excess of free light chains it may be expected that they will appear eventually in the urine where they can both aid in diagnosis of a B cell tumour and also provide an index of tumour load (Pierson *et al.*, 1980).

Methods used previously to examine urine for tumour-related light chains have relied on changes in the levels of one of the light chain types. Results then have to be interpreted against a wide range of κ/λ ratios in normal subjects. Using such a procedure, significant increases in the level of one of the urinary light chain types were seen in 19 out of 76 cases of lymphoma and leukaemia (Lindström *et al.*, 1969).

The presence of such monoclonal light chain in urine from patients with B cell tumours has been

**Table II** Patients with non-Hodgkin's lymphoma

| *Patient* | *Age (yr)* | *Sex* | *Histology on biopsy** | *Stage†* | *Treatment* | *Surface Ig on tumour cells‡* | *Urinary monoclonal light chain* |
|---|---|---|---|---|---|---|---|
| V.E. | 37 | F | CB-CC diffuse | IV | + | λ | ND§ |
| K.G. | 36 | M | CB-CC follicular | II | – | – | +κ |
| M.H. | 39 | M | CB-CC diffuse | I | – | κ | +κ |
| N.B. | 65 | M | CB-CC follicular | II | – | Mκ | ND |
| A.C. | 71 | F | CC follicular | IV | – | – | ND |
| L.R. | 78 | F | CC diffuse | III | + | Mλ | ND |
| E.K. | 57 | M | CC follicular | IV | + | – | +κ |
| K.H. | 64 | F | CB-CC follicular | I | – | – | ND |
| M.D. | 49 | F | CB-CC follicular | IV | + | Mκ | +κ |
| J.H. | 62 | F | CB-CC follicular | III | – | MDκ | +κ |
| M.J. | 63 | F | CB-CC follicular | IV | – | – | ND |
| E.S. | 56 | F | CB diffuse | I | + | Gκ | +κ |
| C.S. | 61 | M | Small cell lymphocytic | IV | – | MGκ | +κ |
| B.S. | 51 | F | Small cell lymphocytic | III | – | λ | ND |
| E.D. | 49 | F | Small cell lymphocytic | I | – | Mλ | ND |
| A.E. | 61 | M | Small cell lymphocytic | IV | + | κ | ND |
| W.W. | 67 | M | Histiocytic | I | – | – | ND |
| P.G. | 43 | M | Histiocytic | III | + | – | ND |
| E.F. | 70 | M | Histiocytic | IV | – | – | ND |
| R.R. | 49 | F | Reactive | – | – | – | ND |
| B.W. | 59 | F | Reactive | – | – | – | ND |
| J.U. | 77 | M | AILD | – | – | – | ND |

*Histological classification was based on the system of Gérard-Marchant *et al.* (1974): CB and CC refer to cells of the follicle centre, centroblasts and centrocytes respectively, with CB-CC indicating a mixture of the 2 cell types. Patterns of tumour cell distribution in the biopsies are indicated as follicular where follicles are recognizable, or diffuse. AILD: angioimmunoblastic lymphadenopathy.

†Staging was according to the Ann Arbor Conference (Carbone, 1971).

‡Data obtained by examination of cell suspensions from fresh tissue biopsies by immunofluorescence. In some cases there was only sufficient material for light chain analysis. Dashes indicate that fresh material was not available.

§ND = not detected.

sought in a previous study from this laboratory and 40–50% of patients with CLL and NHL were found to be positive (Pierson *et al.*, 1980). The method used, however, involved isolation of the light chain by immunosorption and was not applicable on a routine basis. The current report describes a different method of analysis which demonstrates monoclonal light chain by a simple and rapid technique which could be used as a diagnostic aid.

In CLL, where the incidence of urinary monoclonal light chains has been described as 15% when analyzed by conventional electrophoresis or immunoelectrophoresis (McLaughlin & Hobbs, 1972) the techniqe of isoelectric focusing has demonstrated a much higher incidence (14 out of 19 patients studied) suggesting that this immunochemical procedure may assist in diagnosis, particularly when the surface Ig of the neoplastic cells in the blood is scanty.

The incidence of monoclonal urinary light chain in NHL involving B lymphocytes appears to be lower than for CLL (7 out of 16 patients studied). Several factors could account for this: 6 of the patients studied were already on treatment and although 2 of these did have urinary monoclonal light chains, the effect of treatment on the remaining 4 is not known. A further study of patients before treatment is in progress. Another factor is the stage of the disease, as a small body load would not be expected to give rise to detectable light chain: however no obvious correlation with disease stage is apparent from Table II. The third factor is the nature of the tumour cells, in that the more mature B cells may not export excess light chain (Hannam-Harris *et al.*, 1980). Even with these restrictions, the finding of monoclonal light chain in the urine of a patient would be a useful immunochemical adjunct to current diagnostic procedures, although it should be emphasized that failure to detect such a product would not preclude a B cell neoplasm. Histological investigation of biopsy material from patients with suspected NHL, even with analysis of surface Ig, may fail to distinguish the nature of the disease and urine analysis would provide a simple additional test. Also, for both CLL and NHL the load of a tumour-related product in urine would give a measure of total body load and quantitative estimation of urinary light chain e.g. by radioimmunoassay during a course of therapy may be useful in monitoring patients.

One problem which could arise is that of "idiopathic" Bence-Jones proteinuria, particularly when using such a sensitive method of analysis. The existence of such a state has been questioned recently (Kyle & Greipp, 1982) since of 7 patients studied, 5 subsequently demonstrated myeloma although the time to clinical disease was long and variable. Further following of patients with urinary monoclonal light chains should clarify this point.

It is difficult to prove in each case that the urinary light chains have arisen from the tumour cells, although where the surface Ig of the tumour cells has been identified, the light chain in the urine has been of the same type (13 CLL and 5 NHL). However, in 2 of the patients with CLL it has been shown by examination by IEF of culture fluids obtained from isolated cells that the neoplastic cells were the source of the urinary light chains.

A further extension of these findings is that isolation of monoclonal light chains from selected patients can be used to raise anti-light chain isotypes (Pierson *et al.*, 1980). If these react with idiotypic determinants on the tumour cells as has been demonstrated for a B lymphocytic neoplasm of guinea pigs (Stevenson *et al.*, 1977), it may be possible to use such antibody in analysis of tumour distribution and possible immunotherapy.

We are grateful to Dr. T.J. Hamblin and Dr. F. Macbeth for providing clinical details and samples from patients. This work was supported by the Leukaemia Research Fund and the Wessex Regional Health Authority.

### References

BÖYUM, A. (1974). Separation of blood leucocytes, granulocytes and lymphocytes. *Tissue Antigens*, **4**, 269.

CARBONE, P.C. (1971). Report of the Committee on Hodgkin's Disease Staging Classification. *Cancer Res.*, **31**, 1860.

GÈRARD-MARCHANT, R., HAMLIN, I., LENNERT, K., RILKE, F., STANSFELD, A.G. & VAN UNNIK, J.A.M. (1974). Classification of non-Hodgkin's lymphomas. *Lancet*, **ii**, 406.

GORDON, J., HOWLETT, A.R. & SMITH, J.L. (1978). Free light chain synthesis by neoplastic cells in chronic lymphocytic leukaemia and non-Hodgkin's lymphoma. *Immunology*, **34**, 397.

GREY, H.M., RABELLINO, E. & PIROFSKY, B. (1971). Immunoglobulin on the surface of lymphocytes. IV Distribution in hypogammaglobulinaemia, cellular immune deficiency and chronic lymphatic leukaemia. *J. Clin. Invest.*, **50**, 2368.

HANNAM-HARRIS, A.C., GORDON, J. & SMITH, J.L. (1980). Immunoglobulin synthesis by neoplastic B lymphocytes: free light chain synthesis as a marker of B cell differentiation. *J. Immunol.*, **125**, 2177.

HEMMINGSEN, L. & SKAARUP, P. (1975). The 24-hour excretion of plasma protein in the urine of apparently healthy subjects. *Scand. J. Clin. Lab. Invest.*, **35**, 347.

ISAACSON, P., WRIGHT, D.H., JUDD, M.A. & MEPHAM, B.L. (1979). Primary gastrointestinal lymphomas: a classification of 66 cases. *Cancer*, **43**, 1805.

KYLE, R.A. & GREIPP, P.R. (1982). "Idiopathic" Bence Jones proteinuria. Long-term follow-up in seven patients. *N. Engl. J. Med.*, **306**, 564.

LINDSTRÖM, F.D., WILLIAMS, R.C. & THEOLOGIDES, A. (1969). Urinary light chain excretion in leukaemia and lymphoma. *Clin. Exp. Immunol.*, **5**, 83.

LUKES, R.J. & TINDLE, B.H. (1975). Immunoblastic lymphadenopathy. A hyperimmune entity resembling Hodgkin's disease. *N. Engl. J. Med.*, **292**, 1.

MAINO, V.C., KURNICK, J.T., KUBO, R.T. & GREY, H.M. (1977). Mitogen activation of human chronic lymphatic leukaemia cells. I. Synthesis and secretion of immunoglobulin. *J. Immunol.*, **118**, 742.

McLAUGHLIN, J. & HOBBS, J.R. (1972). Clinical significance of Bence–Jones Proteinuria. In *Protides of the Biological Fluids, 20th Colloquim*. (Ed. Peeters). 0. 251.

PIERSON, J., DARLEY, T., STEVENSON, G.T. & VIRJI, M. (1980). Monoclonal immunoglobulin light chains in urine of patients with lymphoma. *Br. J. Cancer*, **41**, 681.

POULIK, M.D. (1964). Heterogeneity of the L(B) chains of gammaglobulins. *Nature*, **202**, 1174.

RAI, K.R., SAWITSKY, A., CRONKITE, E.P., CHANANA, A.D., LEVY, R.N. & PASTERNACK, B.S. (1975). Clinical staging of chronic lymphocytic leukaemia. *Blood*, **46**, 219.

STEVENSON, F.K., ELLIOTT, E.V. & STEVENSON, G.T. (1977). Some effects on leukaemic B lymphocytes of antibodies to defined regions of their surface immunoglobulin. *Immunology*, **32**, 549.

STEVENSON, F.K., HAMBLIN, T.J., STEVENSON, G.T. & TUTT, A.L. (1980). Extracellular idiotypic immunoglobulin arising from human leukaemic B lymphocytes. *J. Exp. Med.*, **152**, 1484.

STEVENSON, G.T. (1976). Biochemical abnormalities in some human neoplasms. (2) Multiple myeloma and other B cell lymphomas. In *Scientific Foundations of Oncology*, (Eds. Symington & Carter), London: William Heinemann. p. 85.